# Subsampled Directed-Percolation Models Explain Scaling Relations Experimentally Observed in the Brain

**DOI:** 10.3389/fncir.2020.576727

**Published:** 2021-01-15

**Authors:** Tawan T. A. Carvalho, Antonio J. Fontenele, Mauricio Girardi-Schappo, Thaís Feliciano, Leandro A. A. Aguiar, Thais P. L. Silva, Nivaldo A. P. de Vasconcelos, Pedro V. Carelli, Mauro Copelli

**Affiliations:** ^1^Departamento de Física, Universidade Federal de Pernambuco, Recife, Brazil; ^2^Department of Physics, University of Ottawa, Ottawa, ON, Canada; ^3^Departamento de Física, Faculdade de Filosofia, Ciências e Letras de Ribeirão Preto, Universidade de São Paulo, Ribeirão Preto, Brazil; ^4^Departamento de Ciências Fundamentais e Sociais, Universidade Federal da Paraíba, Areia, Brazil; ^5^Life and Health Sciences Research Institute (ICVS), School of Medicine, University of Minho, Braga, Portugal; ^6^Life and Health Sciences Research Institute/Biomaterials, Biodegradables and Biomimetics, Braga, Portugal

**Keywords:** subsampling, neuronal avalanches, brain criticality, scaling relations, cortex, urethane

## Abstract

Recent experimental results on spike avalanches measured in the urethane-anesthetized rat cortex have revealed scaling relations that indicate a phase transition at a specific level of cortical firing rate variability. The scaling relations point to critical exponents whose values differ from those of a branching process, which has been the canonical model employed to understand brain criticality. This suggested that a different model, with a different phase transition, might be required to explain the data. Here we show that this is not necessarily the case. By employing two different models belonging to the same universality class as the branching process (mean-field directed percolation) and treating the simulation data exactly like experimental data, we reproduce most of the experimental results. We find that subsampling the model and adjusting the time bin used to define avalanches (as done with experimental data) are sufficient ingredients to change the apparent exponents of the critical point. Moreover, experimental data is only reproduced within a very narrow range in parameter space around the phase transition.

## 1. Introduction

In the first results that fueled the critical brain hypothesis, Beggs and Plenz (2003) observed intermittent bursts of local field potentials (LFPs) in *in vitro* multielectrode recordings of cultured and acute slices of the rat brain. Events occurred with a clear separation of time scales, and were named neuronal avalanches.

A neuronal avalanche can be characterized by its size *S*, which is the total number of significant voltage deflections recorded by electrodes between periods of silence, and by its duration *T*, which is the number of consecutive time bins spanned by an avalanche. Beggs and Plenz found power-law distributions for the sizes of avalanches,

(1) $$\begin{array}{l} P\left(S\right)\sim S^{-\tau }, \end{array}$$

with τ ≃ 3/2, and suggested, based on their data, a power-law distribution of avalanche duration,

(2) $$\begin{array}{l} P\left(T\right)\sim T^{-\tau_{t}}, \end{array}$$

with τ_*t*_ = 2. These scale-invariant distributions were interpreted as a signature that the brain could be operating at criticality—a second-order phase transition (Beggs and Plenz, 2003; Beggs, 2007; Chialvo, 2010; Shew and Plenz, 2013; Plenz and Niebur, 2014; Tomen et al., 2019).

In particular, these two critical exponents together are compatible with a branching process at its critical point (Harris, 1963), a conclusion that was further strengthened by the experimentally established critical branching parameter of 1 for neuronal avalanches (Beggs and Plenz, 2003). This points to a phase transition between a so-called absorbing phase (zero population firing rate) and an active phase (non-zero stationary population firing rate).

Due to its appeal, simplicity, and familiarity within the statistical physics community, the critical branching process has become a canonical model for understanding criticality in the brain. In fact, these exponents are compatible with a larger class of models, namely, any model belonging to the mean-field directed percolation (MF-DP) universality class (Muñoz et al., 1999). In the theory of critical phenomena, two models which can be different in their details are said to belong to the same universality class when the critical exponents which characterize their phase transition coincide (Binney et al., 1992). In general, probabilistic contagion-like models which have a unique absorbing state (all sites “susceptible” or in the neuroscience context, all neurons quiescent) and no further symmetries tend to belong to the directed-percolation universality class (Janssen, 1981; Grassberger, 1982; Marro and Dickman, 1999). If the network has topological dimension above 4 (such as random or complete graphs), the model usually belongs to the MF-DP universality class.

More recently, these ideas were tested with more advanced experimental techniques, highlighting the prospect of criticality in the awake brain. Two studies have shown that different types of anesthesia strongly affect avalanche statistics. In voltage imaging recordings of the mouse cortex, size distributions were more closely compatible with τ = 1.5 for awake animals than for animals anesthetized with pentobarbital (Scott et al., 2014). A similar trend was observed in two-photon imaging of the rat cortex, where avalanche distributions become increasingly compatible with τ = 1.5 and τ_*t*_ = 2 as the animals recover from isoflurane anesthesia (Bellay et al., 2015).

Other experimental results, however, challenged the MF-DP scenario originally proposed by Beggs and Plenz (2003). For instance, avalanche exponents in *ex-vivo* recordings of the turtle visual cortex deviated significantly from τ = 3/2 and τ_*t*_ = 2 (Shew et al., 2015). Discrepancies in exponent values were also observed in spike avalanches of rats under ketamine-xylazine anesthesia (Ribeiro et al., 2010) and M/EEG avalanches in resting or behaving humans (Palva et al., 2013; Zhigalov et al., 2015), among others.

Furthermore, Touboul and Destexhe (2017) argued that the power-law signature alone in the distributions of size (Equation 1) and duration (Equation 2) of avalanches is insufficient to claim criticality, since power laws can be observed in non-critical models as well. They suggested that another scaling relation should be tested as a stronger criterion. This was based on the result that at criticality the average avalanche size 〈*S*〉 for a given duration *T* must obey

(3) $$\begin{array}{l} \langle S\rangle \sim T^{\frac{1}{\sigma \nu z}}, \end{array}$$

where 1/(σ*νz*) is a combination of critical exponents that at criticality satisfy the so-called crackling noise scaling relation (Muñoz et al., 1999; Sethna et al., 2001; Friedman et al., 2012)

(4) $$\begin{array}{l} \frac{1}{\sigma \nu z}=\frac{\tau_{t}-1}{\tau -1}. \end{array}$$

Equation (4) is a stronger criterion for criticality because it is expected not to be satisfied by non-critical models (Touboul and Destexhe, 2017). In the MF-DP case, the avalanche exponents obey (τ_*t*_ − 1)/(τ − 1) = 2 and 1/(σ*νz*) = 2, independently. The absolute difference between the two sides of Equation (4) can even be employed as a metric for the distance to criticality (Ma et al., 2019), or to identify criticality in more general phase transitions of neuronal networks (Girardi-Schappo and Tragtenberg, 2018). Indeed, Ponce-Alvarez et al. (2018) have investigated the crackling noise relation in zebrafish whole-brain activity, obtaining 1/(σ*νz*) ≃ 2 but values of τ and τ_*t*_ incompatible with MF-DP. Miller et al. (2019) have also found 1/(σ*νz*) ≃ 2 in LFP avalanches from awake non-human primates, when the impact of ongoing gamma-oscillations was accurately taken into account.

Recently, cortical spike avalanches of urethane-anesthetized rats were investigated under this methodological lens by Fontenele et al. (2019). This experimental setup is known to yield spiking activity which is highly variable, ranging from very asynchronous to very synchronous population activity (Clement et al., 2008). These regimes can be characterized by different ranges of the coefficient of variation (*CV*) of the population firing rate (de Vasconcelos et al., 2017), which is thought of as a simple marker of cortical states (Harris and Thiele, 2011). By parsing the data according to levels of spiking variability, Fontenele et al. (2019) found that the scaling relation Equation (4) was satisfied at an intermediate value of *CV*, suggesting a phase transition away from both the synchronized and desynchronized ends of the spiking variability spectrum. In particular, the values of the avalanche exponents where the scaling relation was satisfied were not all compatible (within error bars) with MF-DP values: 〈τ〉 ≃ 1.52 ± 0.09, 〈τ_*t*_〉 ≃ 1.7 ± 0.1 and 〈1/(σ*νz*)〉 ≃ 1.28 ± 0.03 (Fontenele et al., 2019). This was interpreted as an incompatibility with the theoretical MF-DP scenario (Fontenele et al., 2019), thus requiring the formulation of models belonging to other universality classes and undergoing other phase transitions.

One hypothesis to explain this controversy is that the study of spike activity is strongly affected by subsampling effects, that is, the measured activity is based on a tiny fraction of the total number of neurons in a given area of the brain. Different groups have shown that subsampling indeed affects the apparent distribution of avalanches (Priesemann et al., 2009, 2014; Ribeiro et al., 2010, 2014; Girardi-Schappo et al., 2013; Levina and Priesemann, 2017; Wilting and Priesemann, 2019). For example, an avalanche evaluated on all elements (full sampling) can be broken into smaller avalanches when recorded in a subset of the network (subsampling). In addition, we highlight that this effect is different from the well-studied phenomenon of finite-size scaling, which is the study of how statistical properties change as the size of the system increases and activity recorded in all sites is analyzed (see e.g., Levina and Priesemann, 2017).

Here, we revisit this issue by studying the data produced by two theoretical models in the MF-DP universality class. We start by showing that the models reproduce well-known analytical results, which however fail to reproduce the experimental data. Then we proceed to treat the model results under the same conditions as those of experimental data. Despite the large number of simulated neurons (~ 10^5^), we intentionally restrict the theoretical analysis to a small subset of cells (~ 10^2^), mimicking the fact that one can only record a few hundred neurons among the millions that comprise the rat's brain (the subsampling issue). Here we show that by combining the subsampling of the model with the analysis pipeline that has been applied to the experimental data (Fontenele et al., 2019), we can reconcile the empirical power-law avalanches with the theoretical MF-DP universality class.

## 2. Methods

### 2.1. A Spiking Neuronal Network With Excitation and Inhibition

We used the excitatory/inhibitory network of Girardi-Schappo et al. (2020), where each neuron is a stochastic leaky integrate-and-fire unit with discrete time step equal to 1 ms, connected in an all-to-all graph. A binary variable indicates if the neuron fired [*X*(*t*) = 1] or not [*X*(*t*) = 0]. The membrane potential of each cell *i* in either the excitatory (*E*) or inhibitory (*I*) population is given by

(5) $$\begin{array}{c} \overset{E/I}{\underset{i}{V}}(t+1)=[\mu \overset{E/I}{\underset{i}{V}}(t)+I_{e}+\frac{J}{N}\overset{j=1}{\underset{N_{E}}{\sum^{\text{​}}}}X_{j}^{E}(t)\\ \;-\frac{gJ}{N}\overset{j=1}{\underset{N_{I}}{\sum^{\text{​}}}}X_{j}^{I}(t)]\left(1-X_{i}^{E/I}(t)\right), \end{array}$$

where *J* is the synaptic coupling strength, *g* is the inhibition to excitation (E/I) coupling strength ratio, μ is the leak time constant, and *I*_*e*_ is an external current. The total number of neurons in the network is $N=N_{E}+N_{I}=10^{5}$, where the fractions of excitatory and inhibitory neurons are kept fixed at *p* = *N*_*E*_/*N* = 0.8 and *q* = *N*_*I*_/*N* = 0.2, respectively, as reported for cortical data (Somogyi et al., 1998). Note that the membrane potential is reset to zero in the time step following a spike.

At any time step, a neuron fires according to a piecewise linear sigmoidal probability Φ(*V*),

(6) $$\begin{array}{l} \Phi \left(V\right)\equiv P\left(X=1|V\right)=\Gamma \left(V-\theta \right)\Theta \left(V-\theta \right)\Theta \left(V_{S}-V\right)+\Theta \left(V-V_{S}\right), \end{array}$$

where θ = 1 is the firing threshold, Γ is the firing gain constant, *V*_*S*_ = 1/Γ + θ is the saturation potential, and Θ(*x* > 0) = 1 (zero otherwise) is the step function. For simplicity, the parameter μ = 0 is chosen without lack of generality, since it does not change the phase transition of the model (Girardi-Schappo et al., 2020). The external current *I*_*e*_ > *V*_*S*_ is used only to spark a new avalanche in a single excitatory neuron when the network activity dies off (it is kept as *I*_*e*_ = θ otherwise).

This model is known to present a directed percolation critical point (Girardi-Schappo et al., 2020) at *g*_*c*_ = *p*/*q* − 1/(*q*Γ*J*) = 1.5 (for Γ = 0.2 and *J* = 10), such that *g* < *g*_*c*_ is the active excitation-dominated (supercritical) phase and *g* > *g*_*c*_ corresponds to the inhibition-dominated absorbing state (subcritical). The synapses in the critical point *g*_*c*_ are dynamically balanced: fluctuations in excitation are immediately followed by counter fluctuations in inhibition (Girardi-Schappo et al., 2020). The initial condition of the simulations has all neurons quiescent except for a seed neuron to spark activity. This procedure was repeated whenever the system went back to the absorbing state.

### 2.2. Probabilistic Cellular Automaton Model

The spiking model described in section 2.1 is certainly not the simplest model to present a phase transition in the MF-DP universality class. Therefore, to probe the robustness of our findings, we also simulated a much simpler model: a network of probabilistic excitable cellular automata in a random graph (Kinouchi and Copelli, 2006). This model closely resembles a standard branching process and is known to mimic the changing inhibition-excitation levels of cortical cultures (Shew et al., 2009).

Each site *i* (*i* = 1, …, *N*) has five states: the silent state, *s*_*i*_ = 0, the active state, *s*_*i*_ = 1, corresponding to a spike, and the remaining three states, *s*_*i*_ = 2, 3, 4, in which the site will not respond to incoming stimuli (absolute refractory states). Each site receives input from *K* presynaptic neighbors which are randomly selected at the start and kept fixed throughout the simulations. A quiescent site *i* becomes excited [*s*_*i*_(*t*) = 0 → *s*_*i*_(*t* + 1) = 1] with probability *p*_*ij*_ if a presynaptic neighbor *j* is active at time *t*. All presynaptic neighbors are swept and independently considered at each time step, so that

(7) $$\begin{array}{l} P\left(s_{i}\left(t+1\right)=1|s_{i}\left(t\right)=0\right)=1-\left(1-h_{i}\right)\overset{K}{\underset{j\in N\left(i\right)}{\prod }}\left[1-p_{ij}s_{j}\left(t\right)\right], \end{array}$$

where *h*_*i*_ is the probability of unit *i* spiking due to an external stimulus and N(i) is the set of presynaptic neighbors of *i*. The remaining transitions happen with probability 1, including the transition 4 → 0 that returns the site to its initial quiescent state. The time step of the model corresponds to 1 ms.

We initially chose the random variables {*p*_*ij*_} from a uniform distribution in the interval [0, 2λ/*K*]. The so-called branching ratio λ = *K*〈*p*_*ij*_〉 is the control parameter of the model. This model undergoes a MF-DP phase transition at λ = λ_*c*_ = 1 (Kinouchi and Copelli, 2006). For λ < 1, the system is in the subcritical phase and eventually reaches the absorbing state (*s*_*i*_ = 0, ∀*i*). For λ > 1, the system presents self-sustained activity, i.e., a non-zero stationary density of population firings (the supercritical phase). The critical point is not affected by the number of refractory states (Kinouchi and Copelli, 2006).

In our simulations we used *K* = 10 neighbors for each of the *N* = 10^5^ sites. Similarly to the spiking neuronal network model, a single random neuron was stimulated (*h*_*i*_ = 1) only when the system reached the absorbing state, sparking the network activity and subsequently being set back to *h*_*i*_ = 0. The initial condition was set with a single randomly chosen site active and the others in the silent state.

### 2.3. Experimental Data Acquisition

Urethane is a well-established drug that provides spontaneous changes of brain states that resemble sleep state alternations (Clement et al., 2008). In the last decade, experimental preparations using urethane have helped elucidate questions concerning mechanisms and the functional relevance of state-dependent patterns of brain activity (Curto et al., 2009; Renart et al., 2010; Mochol et al., 2015; de Vasconcelos et al., 2017). The property to promote spontaneous change in the levels of spiking variability cannot be achieved in other anesthesia approaches, such as pentobarbital and isoflurane.

The data used in this paper is original and corroborates the results of Fontenele et al. (2019). We used five rats Long-Evans (*Rattus norvegicus*) (male, 280–360 g, 2–4 months old). They were obtained from the animal house of the Laboratory of Computational and Systems Neuroscience, Department of Physics, Federal University of Pernambuco (UFPE). The animals were anesthetized with urethane (1.55 g/kg), diluted at 20% in saline, in three intraperitoneal (i.p.) injections, 15 min apart (Sakata and Harris, 2009). Some animals demanded supplement (max 5%) of urethane to reach the proper level of analgesia. In order to ensure that the animals are maintained at the correct depth of anesthesia, responses to painful stimuli (pinching the animal's toes, ears and tail) were always checked throughout the experiment. Once the anesthesia reached its proper level, the rats were placed in a stereotaxic frame and the coordinates to access the primary visual cortex (V1) were marked (Bregma: AP = −7.2, ML = 3.5) (Paxinos and Watson, 2007). A cranial window in the scalp was opened using this coordinate as center, with an area of ~3 mm^2^. We performed recordings of extra-cellular voltage of neuronal populations by using a 64-channel multielectrode silicon probe (Neuronexus technologies, Buzsaki64spL-A64). This probe has 60 electrodes disposed in six shanks separated by 200 μm, 10 electrodes per shank with impedance of 1–3 MOhm at 1 kHz. Each electrode has 160 μm^2^ and they are in staggered positions 20 μm apart. We recorded from deeper layers of the rat cortex, similarly to what was previously done in Ribeiro et al. (2010) under ketamine-xylazine and Fontenele et al. (2019) under a setup similar to the one presented here.

Data was sampled at 30 KHz, amplified and digitized in a single head-stage (Intan RHD2164) (Siegle et al., 2017). We recorded spontaneous activity, during long periods (≥3 h). We used the open-source software Klusta to perform the automatic spike sorting on raw electrophysiological data (Rossant et al., 2016). The automatic part is divided in two major steps, spike detection and automatic clustering. The first step detects action potentials and the second one arrange those spikes into clusters according to their similarities (waveforms, PCA, refractory period). After the automatic part, all formed clusters are reanalyzed using the graphic interface phy kwikGUI^1^. Manual spike sorting allows the identification of each cluster of neuronal activity as single-unit activity (SUA) or multi-unit activity (MUA). We used both SUA and MUA clusters for our study.

### 2.4. Avalanche Analysis With CV Parsing

To study neuronal avalanches at different levels of spiking variability (Shadlen and Newsome, 1998), we segmented both the neurophysiological and simulated data in non-overlapping windows of width *w* = 10 s (unless otherwise stated) (Gervasoni et al., 2004). Each of these 10 s epochs was subdivided in non-overlapping intervals {ζ_*j*_} of duration Δ*T* = 50 ms (unless otherwise stated) in which we estimated the population spike-count rate *R*_*j*_. We then calculated the coefficient of variation (*CV*) for the *i*-th 10 s window:

(8) $$\begin{array}{l} CV_{i}=\frac{\sigma_{i}}{\mu_{i}}, \end{array}$$

where *CV* is dimensionless, and σ_*i*_ and μ_*i*_ correspond to the standard deviation and the mean of {*R*_*j*_}, respectively.

For each 10 s window with a particular *CV* level, we proceeded with the standard avalanche analysis of Beggs and Plenz (2003). The summed population activity was sliced in non-overlapping temporal bins of width Δ*t* = 〈*ISI*〉 (the average inter-spike interval). Ribeiro et al. (2010) and Yu et al. (2017) have shown that an adaptive bin, evaluated according to the current dynamical state, renders signatures of scale-free dynamics more robust. Following this strategy, we have separately computed Δ*t* = 〈*ISI*〉 for each 10 s window. Population spikes preceded and followed by silence define a spike avalanche. The number of spikes correspond to the avalanche size *S*, whereas the number of time bins spanned by the avalanche is its duration *T*. Following this methodology, we associated each 10 s *CV*_*i*_ window with its corresponding set of *n*_*i*_ avalanche sizes **S_i_** ≡ {*S*_*i*1_, *S*_*i*2_, …, *S*_*i*_*n*__*i*__} and durations **T_i_** ≡ {*T*_*i*1_, *T*_*i*2_, …, *T*_*i*_*n*__*i*__}.

To estimate the avalanche exponents τ and τ_*t*_, we first ranked the sets {**S_i_**} and {**T_i_**} according to their *CV* values. Next, in order to increase the number of samples while preserving the level of spiking variability, we pooled *NB* consecutive ranked blocks of similar *CV* values (*NB* = 50 unless otherwise stated). For each set of *NB* blocks we calculated the average coefficient of variation 〈*CV*〉. The exponents of the size and duration distributions were obtained via a Maximum Likelihood Estimator (MLE) procedure (Deluca and Corral, 2013; Yu et al., 2014; Marshall et al., 2016) on a discrete power-law distribution

(9) $$\begin{array}{l} f\left(x\right)=\frac{1}{\sum_{x=x_{min}}^{x_{max}}{\left(\frac{1}{x}\right)}^{\alpha }}{\left(\frac{1}{x}\right)}^{\alpha }. \end{array}$$

The standard choice of fitting parameters, for both experimental and subsampled simulated data, was *S*_*min*_ = 2 and *S*_*max*_ = 100 for size distributions and *T*_*min*_ = 2 and *T*_*max*_ = 30 for duration distributions. The exceptions to this choice were for the data shown in **Figures 4C,D**, due to a change of orders of magnitude in the number of neurons sampled. The specific parameters for these cases are shown in Table 1.

**Table 1:** Limits chosen for the calculation of the α exponent (Equation 9) via Maximum Likelihood Estimator (MLE) only for the model data shown in Figures 4C,D (Δ*t* = 1 ms).

***n***	**Size distribution**		**Duration distribution**	
	***S*_*min*_**	***S*_*max*_**	***T*_*min*_**	***T*_*max*_**
100	2	30	2	15
200	2	100	2	50
500	2	200	2	70
1,000	2	200	2	70
2,000	2	300	3	100
5,000	2	500	4	100
10,000	5	3,000	5	150
20,000	5	5,000	5	200
30,000	10	10,000	10	200
40,000	10	10,000	10	250
50,000	10	10,000	10	300
100,000	10	20,000	10	300

*See text for details*.

After the MLE fit we used the Akaike Information Criterion (*AIC*) as a measure of the relative quality of a given statistical model for a data set:

(10) $$\begin{array}{l} AIC=2k-2\ln\left(\hat{L}\right)+\frac{2k^{2}+2k}{N-k-1}, \end{array}$$

where $\hat{L}$ is the likelihood at its maximum, *k* is number of parameters and *N* the sample size (Akaike, 1975). Starting from the principle that lower *AIC* indicates a more parsimonious model, we defined Δ ≡ *AIC*_*ln*_ − *AIC*_*pl*_, where *AIC*_*ln*_ and *AIC*_*pl*_ correspond to the *AIC* of a log-normal and a power-law model, respectively. Therefore, Δ > 0 implies that a power-law model is a better fit to the data than a log-normal. Our scaling relation analyses were restricted to distributions that satisfied Δ > 0.

### 2.5. Pairwise Correlations

Pairwise spiking correlations were estimated using only the SUA or the simulated data in the following way: first, for each cell *k* we obtained a spike count time series *R*^(*k*)^(*t*) at millisecond resolution (Δ*T* = 1 ms), then each spike count time series *R*^(*k*)^ was convolved with a kernel *h*_*t*_1_,*t*_2__(*t*) to estimate the *k*-th mean firing rate *n*^(*k*)^(*t*):

(11) $$\begin{array}{l} n^{\left(k\right)}\left(t\right)=h_{t_{1},t_{2}}\left(t\right)*R^{\left(k\right)}\left(t\right), \end{array}$$

where *h*_*t*_1_,*t*_2__(*t*) is a Mexican-hat kernel obtained by the difference between zero-mean Gaussians with standard deviations *t*_1_ = 100 ms and *t*_2_ = 400 ms (Renart et al., 2010). The *n*_*k*_(*t*) were employed to calculate the spiking correlation coefficient between two units *k* and *l*:

(12) $$\begin{array}{l} r^{\left(k,l\right)}=\frac{\text{Cov}\left(n^{\left(k\right)},n^{\left(l\right)}\right)}{\sqrt{\text{Var}\left(n^{\left(k\right)}\right)\text{Var}\left(n^{\left(l\right)}\right)}}, \end{array}$$

where Var and Cov are the variance and covariance over *t*, respectively.

## 3. Results

### 3.1. Avalanches in the Fully Sampled Model

**Figure 1:**
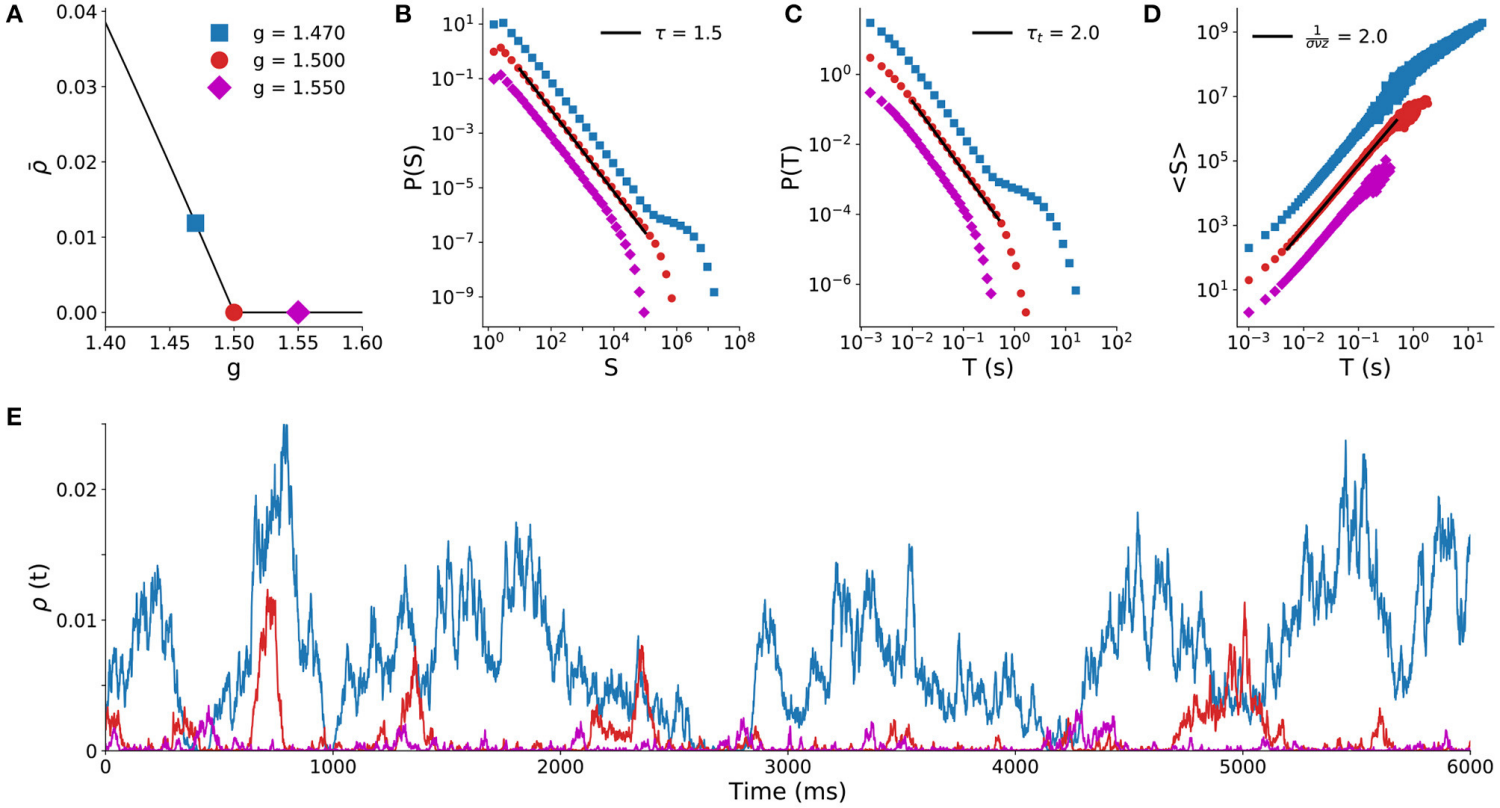
Spiking model results with full sampling. Behavior of the spiking model (*N* = 10^5^) for different values of the control parameter *g*. **(A)** Stationary density of firings $\bar{\rho }$ as a function of the inhibition strength *g* (critical point is the red circle at *g*_*c*_ = 1.5). Solid line is the mean-field solution (Girardi-Schappo et al., 2020), points are simulation results. Distribution of avalanche sizes **(B)** and duration **(C)** for the subcritical (*g* > *g*_*c*_), critical (*g* = *g*_*c*_) and supercritical (*g* < *g*_*c*_) regimes. **(D)** Average avalanche size 〈*S*〉 of a given duration (*T*). **(E)** Time series of the density of active sites for the three regimes.

We start by illustrating the second order phase transition that the model undergoes at a critical value *g*_*c*_ = 1.5 of the inhibition parameter (Girardi-Schappo et al., 2020). As shown in Figure 1A, the stationary density of active sites $\bar{\rho }$ is positive for *g* < *g*_*c*_ (the supercritical regime) and null for *g* > *g*_*c*_ (the subcritical regime).

At the critical point *g* = *g*_*c*_, the distribution of avalanche sizes and duration obey the expected power laws (Equations 1 and 2) with exponents τ = 3/2 and τ_*t*_ = 2 (Girardi-Schappo et al., 2020). Subcritical avalanches are exponentially distributed, whereas the supercritical distribution has a trend to display larger and longer avalanches (Figures 1B,C). Both sides of the scaling law in Equation (4) independently agree, since the fit to 〈*S*〉(*T*) yields 1/(σ*νz*) = 2 on the critical point (Figure 1D). Figure 1E shows typical time series of firing events for the three regimes. These exponents and dynamic behavior of the model are typical of a system undergoing a MF-DP phase transition.

### 3.2. Comparison of Subsampled Model and Experiments Stratified by CV

We now revisit the model by subjecting it to the same constraints that apply to experimental datasets (Fontenele et al., 2019) and compare the results between the two. More specifically: (1) data analysis necessarily uses only a tiny fraction of the total neurons in the system and (2) in urethane-anesthetized rats, cortical spiking variability is a proxy for cortical states (Harris and Thiele, 2011) and changes a lot during the hours-long recordings (Clement et al., 2008; de Vasconcelos et al., 2017).

**Figure 2:**
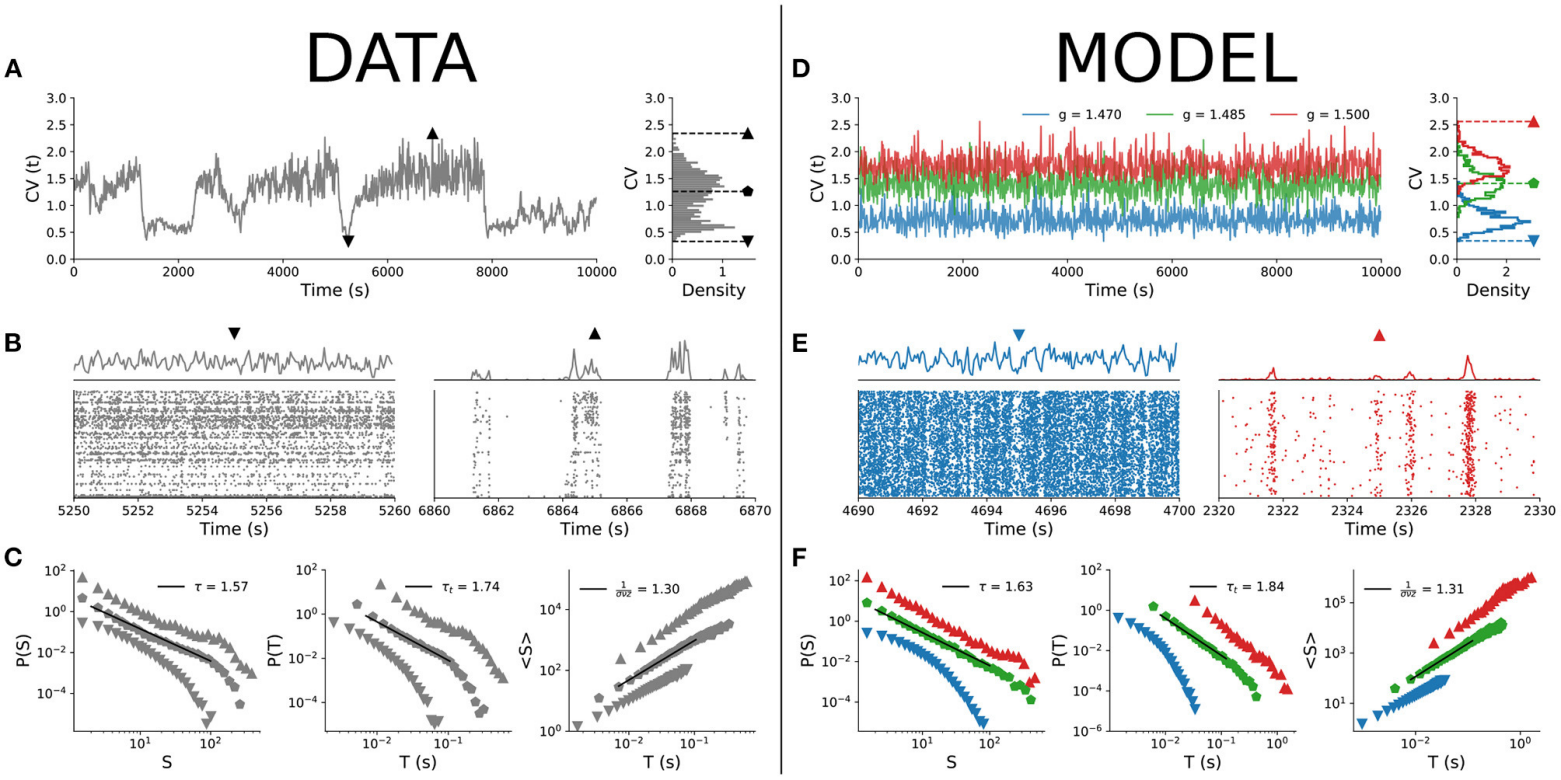
Comparison between empirical data and subsampled spiking model. *CV* time series and distribution for **(A)** experimental data (single animal) and **(D)** model with *n* = 100. Raster plots and population firing rate in cases of low (▼) and high (▲) values of *CV* for **(B)** experimental data and **(E)** model. Scaling exponents τ, τ_*t*_, and 1/(σ*νz*) for three different values of *CV* (denoted by different symbols): **(C)** experimental data and **(F)** model. For both experimental data and model, *w* = 10 s.

Starting with the experimental results, Figure 2A shows the time series of the coefficient of variation (*CV*) of the population spiking activity. The lowest *CV* values correspond to asynchronous spiking activity, whereas the highest values correspond to more synchronized activity (both shown in Figure 2B). When we parsed the data by *CV* percentiles and evaluated neuronal avalanches for different percentiles, the distributions varied accordingly, with exponents τ, τ_*t*_, and 1/(σ*νz*) varying continuously across the *CV* range (Figure 2C) as expected (Fontenele et al., 2019).

Can the MF-DP spiking network model reproduce these experimental results? We found that by sampling only a few neurons out of the entire network, indeed it can. Out of *N* = 10^5^ simulated neurons, we sampled only *n* = 100, a number that is of the same order as the amount of neurons captured in our empirical data (Fontenele et al., 2019). Then, we applied to the subsampled simulation data exactly the same analysis pipeline used for experiments (section 2.4).

In the model, we changed the E/I level *g* to control for the spiking variability level *CV*. For a fixed value of parameter *g*, *CV* is a bell-shaped distribution with finite variance. The *CV*(*t*) time series of the model for a single *g* does not present the dynamical complexity observed experimentally (compare Figures 2A,D). By varying *g* within a narrow interval around the critical point *g*_*c*_, the *CV* distribution of the model covers the values observed experimentally (Figure 2D), with less synchronous behavior for low *CV* and more synchronous activity for high *CV* (Figure 2E; the full behavior of the *CV* distribution as a function of parameter *g* is shown in Supplementary Figure 1A). Parsing the data by *CV* and running the avalanche statistics for the subsampled model, we obtained scaling exponents that vary continuously, in remarkable similarity to what is observed in the experimental data (Figure 2F).

**Figure 3:**
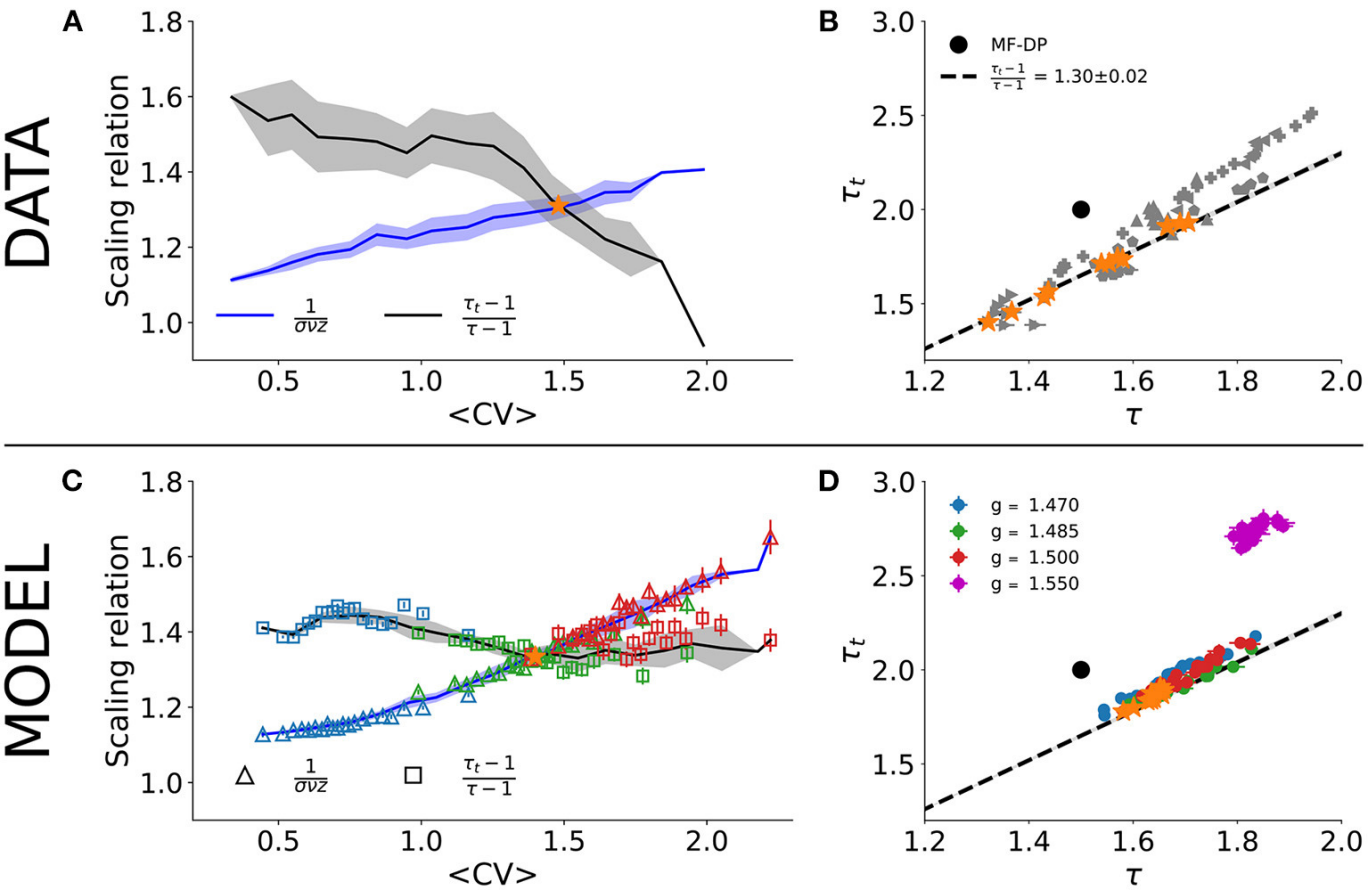
Scaling relation and parametric plot of avalanche exponents. Right- and left-sides of Equation (4) (line and shade are average and standard deviation across the group) as a function of the average *CV* for **(A)** experimental data and **(C)** subsampled model (*n* = 100; note that color code and values of *g* are the same as in Figures 2D,F). Scatter plot in the (τ, τ_*t*_) plane for **(B)** experimental data and **(D)** subsampled model. In both cases, Δ*t* = 〈*ISI*〉 and *w* = 10 s. The star points in **(B,D)** indicate the values of τ and τ_*t*_ that satisfied Equation (4) in **(A,C)**. In Supplementary Figure 2, we show the same result in **(A)** for each rat separately.

A critical system with an absorbing-active phase transition which satisfies Equations (1)–(3) is also expected to satisfy the so-called crackling noise scaling relation of Equation (4). Figure 3A shows the independent experimental fits for the left- and right-hand sides of Equation (4) as a function of *CV*. The crossing at CV_*_ ≃ 1.46±0.08 is consistent with the phase transition reported by Fontenele et al. (2019). In the crossing CV_*_, we obtain τ_*_ = 1.54 ± 0.12, τ_t*_ = 1.73±0.18, and 1/(σ*νz*)_*_ = 1.30 ± 0.02. Plotting τ vs. τ_*t*_, the experimental data scatter along the line with slope given by 1/(σ*νz*)_*_ for different values of *CV* (Figure 3B). These results are in agreement with those of Fontenele et al. (2019), again suggesting an incompatibility with the MF-DP universality class.

The results for the subsampled spiking model, however, suggest otherwise. We did exactly the same procedure with the subsampled model and found a similar *CV* for the crossing of the critical exponents, $CV_{*}^{model}≃1.41\pm 0.05$, when controlling for the E/I ratio *g* very close to the critical point *g*_*c*_ = 1.5 (Figure 3C). On the crossing $CV_{*}^{model}$, we obtained τ_*_ = 1.65 ± 0.02, τ_t*_ = 1.87 ± 0.03, and 1/(σ*νz*)_*_ = 1.34 ± 0.02. Note that these critical exponents are not the true exponents of the model. In fact, they are apparent exponents generated by subsampling the network activity. The true critical exponents are τ = 3/2, τ_*t*_ = 2 and 1/(σ*νz*) = 2 (as shown in Figure 1).

To reproduce the experimental results, the control interval of *g* was slightly biased toward the supercritical range: *g*_*min*_ ≃ 1.47 ≤ *g* ≤ *g*_*max*_ ≃ 1.50. Our model predicts, then, that the whole range of experimental results is produced by fluctuations of only about 2% around the critical point (Figure 3D). For instance, for *g* = 1.55 (3% above the critical point in the subcritical regime), the scaling relation is no longer satisfied and the measured exponents fall far away from the linear relation observed experimentally in the (τ, τ_*t*_) plane (Figure 3D).

This result shows that the MF-DP phase transition under subsampling conditions is capable of reproducing a whole range of experimentally observed avalanches across a range of *CV* values. To test the robustness of our findings, we employed exactly the same procedure to a simpler model, a probabilistic cellular automaton (section 2.2). This model is also knowingly of the MF-DP type (Kinouchi and Copelli, 2006), but has a random network topology. All the results were similar (see Supplementary Figure 3), showing that the apparent exponents are a direct consequence of subsampling.

### 3.3. Dependence on Sampling Fraction and Time Bin Width

**Figure 4:**
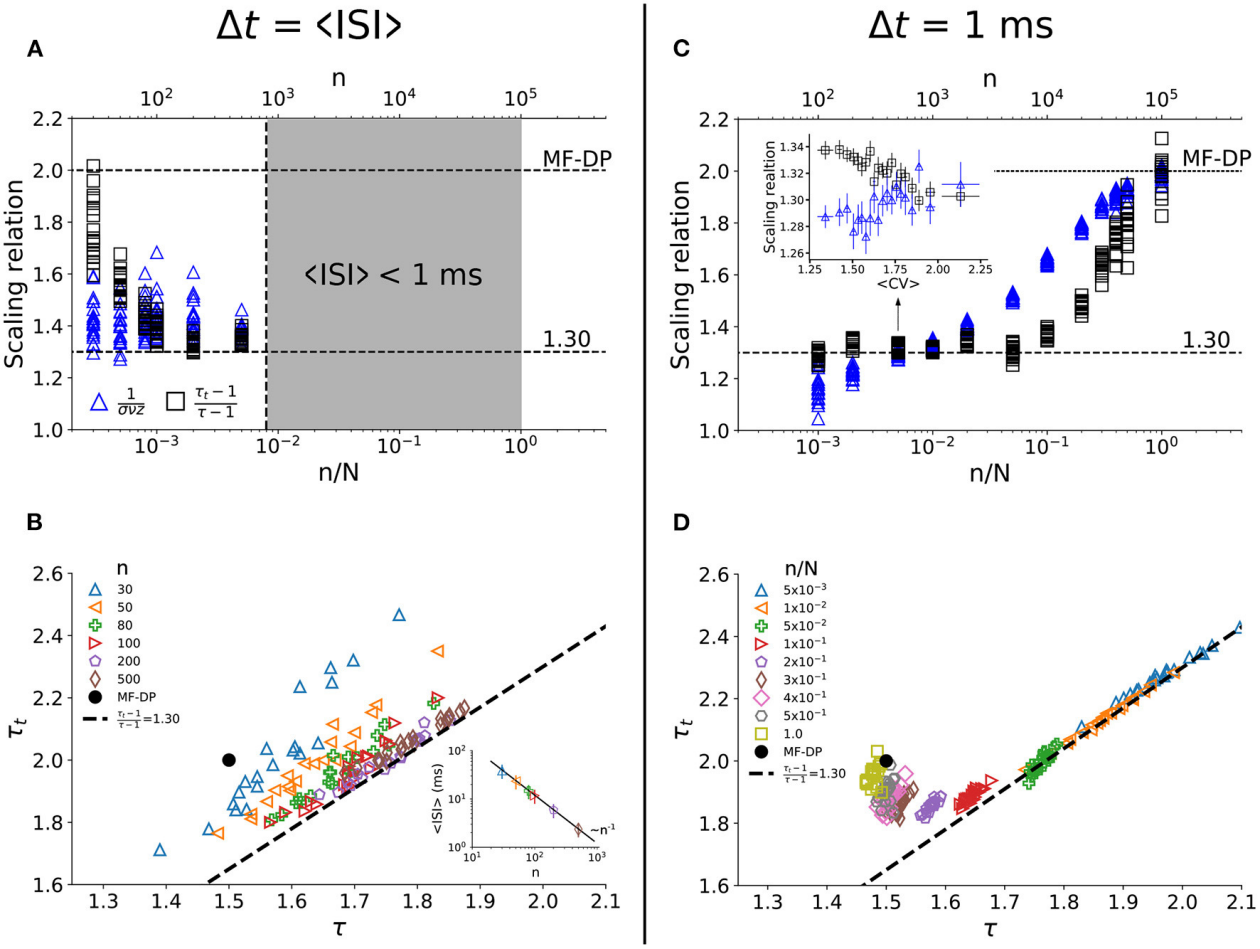
Dependence of the apparent critical exponents on the sampling parameters at criticality. In **(A,C)**, we show both sides of the scaling relation (Equation 4) for *all* values of *CV* observed in the simulations. For each value of *n*/*N*, one has the equivalent of the projection of Figure 3C onto its vertical axis. For Δ*t* = 〈*ISI*〉, **(A)** the scaling relation is satisfied for increasing number of sampled neurons **(B)** with exponents that agree with experimental data. Since 〈*ISI*〉 decreases with *n* [inset of **(B)**], this analysis breaks down when *n* is so large that 〈*ISI*〉 becomes smaller than 1 ms [gray region in **(A)**], which is the time step of the simulations. For Δ*t* = 1 ms, **(C)** the scaling relation is satisfied for small *n*/*N*, within a relatively wide range of *CV* values [inset of **(C)**]. For *n*/*N* → 1, results converge to MF-DP values **(C,D)**, as expected. Simulations with *N* = 10^5^ and *g*_*c*_ = 1.5.

How robust are the results of the model at criticality against variation in the sampling size (*n*) and time bin width (Δ*t*)? First, we considered the time bin width as the population interspike interval Δ*t* = 〈*ISI*〉. The minimum sampling size we employed was *n* = 30 so that power laws still satisfied Akaike's Information Criterion. The agreement of both sides of the scaling law enhances with growing sampling fraction (Figures 4A,B). However, 〈*ISI*〉 decreases with the number of neurons sampled (inset of Figure 4B). When the natural bin decreases below 1 ms (the time step of the model), the analysis no longer makes sense. As *n* increases, the relation between τ and τ_*t*_ converges to the apparent critical scaling that fits experimental results (Figure 4B).

To check whether we could recover the MF-DP real exponents from their apparent values as *n* increases, we chose the smallest time bin possible, Δ*t* = 1 ms. We observed that for a small fraction of sampled units [$n/N\sim O\left(10^{-2}\right)$] the scaling relation (Equation 4) is satisfied (Figure 4C) with apparent critical exponents that match the experimental results (Figure 4D). In fact, the scaling relation in Equation 4 is satisfied for a range of *CV* values (inset of Figure 4C). Increasing the sampling further [$n/N\sim O\left(10^{-1}\right)$], the scaling relation ceases to be satisfied (Figure 4C) and the avalanche exponents get separated from the experimental scaling relation (Figure 4D). But as *n* → *N*, the MF-DP scaling relation is recovered (as it should).

We have further tested the robustness of these findings by varying the time bin width used to defined avalanches (0.75 ≤ Δ*t*/〈*ISI*〉 ≤ 2). We observed that experiments and model have very similar behavior (Supplementary Figures 4A,B). Furthermore, both model and experiments are virtually insensitive to the width of the *CV* window *w* (Supplementary Figures 4C,D). Finally, we also tested whether allowing for small changes of *g* around *g*_*c*_ with *n* = *N* would lead to apparent exponents compatible with experimental data. We observed in this case that the exponents and the scaling relation cluster around MF-DP values (Supplementary Figure 5), reinforcing the idea that subsampling is a necessary ingredient for the model to reproduce the experimental results.

### 3.4. Pairwise Correlation Structure

**Figure 5:**
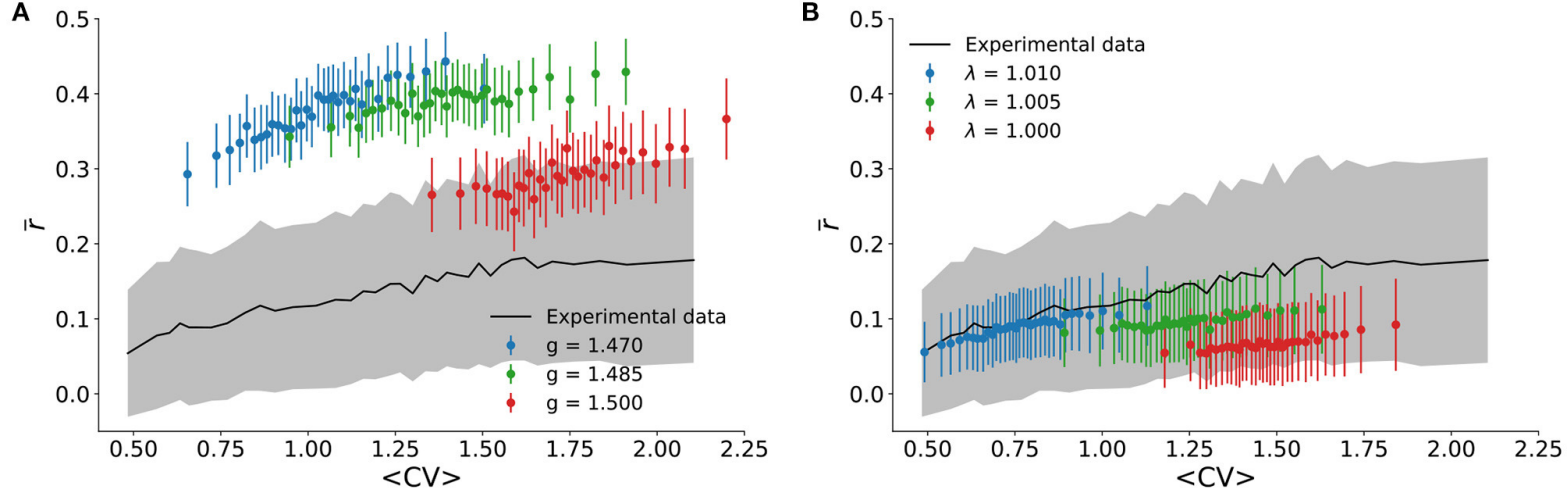
Correlation structure. The experimental pairwise correlation of firing rates is shown as a function of 〈*CV*〉 (black line is the average $\bar{r}$, while gray shading is the standard deviation of the distribution). It is compared with theoretical results for **(A)** the spiking neuronal network with *n* = 100 sampled neurons, and **(B)** the cellular automaton model with *n* = 500 sampled sites.

We also tested the correlation structure of the model and compared it to experimental results. In the literature on cortical states, asynchronous states are associated with pairwise spiking correlations *r*^(*k, l*)^ which are distributed around an average $\bar{r}$ close to zero, whereas synchronous states have positive average (Harris and Thiele, 2011). This was quantified in Figure 5A, where $\bar{r}$ is shown to increase monotonically with *CV*. For the experimental data, $\bar{r}$ reaches zero within the standard deviation of the distribution for sufficiently small *CV*.

Compared with the experimental results, the spiking model with inhibition generally overestimates $\bar{r}$ (Figure 5A). This could be due to its all-to-all connectivity. The cellular automaton model on a random graph yields quantitatively better results (Figure 5B). In either case, we observed again that, just like for the scaling relation (Figure 3), the correlation structure of the experimental data was relatively well-reproduced by very small deviations around critical parameter values.

## 4. Discussion and Conclusions

We revisited the results recently published by Fontenele et al. (2019) by repeating their analyses on new experimental data and two different models. To test the idea that the urethanized cortex hovers around a critical point, we stratified the avalanche analyses across cortical states. For the new experimental data, we verified that the scaling relation combining the three exponents (Equation 4) was indeed satisfied at an intermediate value CV_*_, away from the synchronous and asynchronous extremes. At this critical value, the three exponents differ from those of the MF-DP universality class, thus confirming previous findings (Fontenele et al., 2019).

We addressed whether the exponents of the MF-DP universality class and those observed experimentally could be reconciled, despite their disagreement. In other words, we returned to the question: if the brain is critical, what is the phase transition? Do the experimental results presented here and in Fontenele et al. (2019) refute branching-process-like models as explanations?

To answer these questions, we relied on two models: an E/I spiking neuronal network in an all-to-all graph; and a probabilistic excitable cellular automaton in a random graph. Despite the simplicity and limitations of these models (which we discuss below), they have a fundamental strength that led us to choose them: they are very well-understood analytically. In both cases, mean-field calculations agree extremely well with simulations, so that we are safe in locating the critical points of these models (Kinouchi and Copelli, 2006; Girardi-Schappo et al., 2020). This is very important for our purposes, because it allows us to test whether the models can reproduce the data, and if so, how close to the critical point they have to be. Besides, their universality class is also well-determined: the exponents shown in Figures 1B–D are those of with MF-DP.

The crucial point is that the results in Figure 1 are based on avalanches which are measured by taking into account *all* simulated units of the model, a methodological privilege that is not available to an experimentalist measuring spiking activity of a real brain with current technologies. In fact, a considerable amount of work has shown that subsampling can have a drastic effect on the avalanche statistics of models (Priesemann et al., 2009, 2014; Ribeiro et al., 2010, 2014; Girardi-Schappo et al., 2013; Levina and Priesemann, 2017; Wilting and Priesemann, 2019). Therefore, here we set out to test whether MF-DP models could yield results nominally incompatible with that universality class if they were analyzed under the same conditions as the data, i.e., with *CV* parsing and severe subsampling.

Both subsampled models quantitatively and qualitatively reproduced the central features of the experimental results. The scaling relation (Equation 4) was satisfied at an intermediate value 〈*CV*〉_*_, with the correct qualitative behavior of both sides of the equation: 1/(σ*νz*) increasing with *CV*, while (τ_*t*_ − 1)/(τ − 1) decreasing (see Figures 3A,C and Supplementary Figure 3D). In fact, the values of 〈*CV*〉_*_, and those of the apparent exponents of the subsampled MF-DP models, τ_*_, τ_t*_, and 1/(σ*νz*)_*_, agreed with the experiments within error bars. Moreover, even away from the point 〈*CV*〉_*_ where Equation (4) was satisfied, the spread of the exponents τ and τ_*t*_ of the subsampled models followed an almost linear relation (Figure 3D and Supplementary Figure 3E), in good agreement with not only our experimental results (Figure 3B), but also with those of other experimental setups (Fontenele et al., 2019). When we sampled from the whole network, we recovered the true critical exponents of the model (Figures 4C,D), confirming that spatial subsampling and temporal binning are sufficient ingredients to push its critical exponents toward apparent values, hiding its true critical phase transition.

Knowing analytically the critical points of the models, we checked in which parameter range they successfully reproduced the experimental results. As it turns out, the scaling relation and the linear of spread of exponents are reproduced by the subsampled models only if they are tuned within a narrow interval around their critical points. The subsampled model still fits well the urethanized cortex data up to 3% off criticality, slightly biased toward the supercritical state. Note that if the model becomes too subcritical, the size and duration exponents fall very far apart from the experimentally observed linear relation (Figure 3D). If it is too supercritical, there are not enough silent windows to distinguish avalanches in the first place. Whether or not the fluctuations around the critical point of the model could be compatible with a scenario of self-organized-quasi-criticality (Buendía et al., 2020; Kinouchi et al., 2020) remains to be investigated.

Despite the small variation of the model E/I levels controlled by *g*, the variation of *CV* is large enough to essentially cover the range of experimentally observed values (Figures 2A,D and Supplementary Figure 3B). This is due in part to the fact that we evaluated *CV* within finite windows of width *w* = 10 s. In Supplementary Figure 6 we show that, for the model, the standard deviation of *CV* is a decreasing function of the time used to estimate, all the way up to *w* = 500 s. For the data, on the other hand, a better resolution for *CV* can be obtained by increasing *w* up to about 20 s, above which the standard deviation no longer decreases. It is important to note, however, that in experiments one needs to reach a good trade-off between a better statistical definition of *CV* and not mixing different cortical states due to the non-stationarity characteristic of the urethane preparation (as depicted in Figure 2A).

Perhaps even more important than the range of *CV* values obtained around the critical point of the models is the richness of the experimentally observed temporal evolution of *CV* (Figure 2A). The model needs to be fine tuned to different values of E/I levels in order to get different average values of *CV*. This is one of the limitations of the models which would be worth addressing next. One possibility would be to replace static models (i.e., with fixed control parameters) with ones with plasticity, in which coupling parameters are themselves dynamic variables and the critical point is obtained via quasicritical self-organization (Costa et al., 2015, 2017; Brochini et al., 2016; Campos et al., 2017; Kinouchi et al., 2019, 2020; Buendía et al., 2020; Girardi-Schappo et al., 2020).

Moreover, both models failed to capture the steep drop of (τ_*t*_ − 1)/(τ − 1) as a function of *CV* that is observed in the experimental data above CV_*_ (compare Figure 3A with Figure 3C and Supplementary Figure 3D). This region corresponds to high *CV*, where the models, which are entering their subcritical regimes, seem unable to quantitatively account for the statistics of the increasingly bursty behavior of the data. Whether different models (or a refinement of the ones presented here) could reproduce these results more accurately remains to be studied.

Another limitation of the models is their simple topology, which in future works could be improved to come closer to cortical circuitry (Potjans and Diesmann, 2014). This would likely come at the cost of foregoing analytical results to start with, thus augmenting the computational efforts involved. But it would certainly allow to probe the robustness of the results presented here against more realistic topologies. On the other hand, there is quantitative agreement between the apparent exponents of both models (each having a different topology) with the experimental exponents. This suggests that at the scale of the present phenomenology, the average topology should play a minor role.

It is also interesting to compare the performance of the two models in reproducing the experimental results. The cellular automaton model has the advantage of simplicity, corresponding essentially to a minimal model in the MF-DP universality class. The E/I balanced network, on the other hand, has the advantage of incorporating inhibition, which is an important ingredient for modeling cortical circuitry. As shown in detail in Supplementary Figure 4, the cellular automaton results generally agreed with experimental results, but those of the E/I balanced network had a consistently better agreement. The only exception in this trend was the correlation structure shown in Figure 5, in which the cellular automaton model fared better than the E/I balanced network. In this sense, the models complement each other.

Our model predicts that, for a fixed bin size, increasing the sampling of the data would eventually lead exponents to coincide with those of MF-DP (Figure 4C). However, below a sufficiently high sampling [see, e.g. $n/N\sim O\left(10^{-1}\right)$ in Figure 4C], the scaling relation would not be satisfied for any *CV* even if the system were critical (as the model is). An experimental verification of these predictions would require the recording of a much larger number of neurons than we have presented here.

The fact that subsampling seems to be a crucial ingredient for explaining the data is a double-edged sword. On the one hand, it allowed us here to reconcile MF-DP models with results for spiking data in the anesthetized rat cortex. On the other hand, note that even measurements which should in principle be less prone to subsampling, such as LFP results in the visual cortex of the turtle (Shew et al., 2015), still fall on the same scaling line of τ vs. τ_*t*_ (Figure 3B) as those of spiking data (Fontenele et al., 2019), both having apparent non-MF-DP critical exponents (note, however, that better controlled LFP results in Miller et al., 2019 are in line with MF-DP). This issue is not addressed by the current model and deserves further investigation. Our results point only to MF-DP models as sufficient, not as necessary, to explain the observed phenomenology. So it is at least conceivable that different models with different phase transitions (di Santo et al., 2018; Dalla Porta and Copelli, 2019; Pinto and Copelli, 2019) could also yield non-trivial true or apparent exponents compatible with the data, even without subsampling (Fontenele et al., 2019).

Finally, our simulation results underscore the methodological vulnerabilities of assessing criticality exclusively via avalanche analysis. Not only are MLE power-law fits sensitive to parameters, but even a more stringent analysis, requiring the crackling noise scaling relation, leads to non-trivial apparent exponents which are an artifact of subsampling, as we have shown. Therefore, the development of additional figures of merit, such as control and order parameters, susceptibilities and others (Tagliazucchi et al., 2012; Yang et al., 2012; Yu et al., 2013; Mora et al., 2015; Tkačik et al., 2015; Girardi-Schappo et al., 2016; Girardi-Schappo and Tragtenberg, 2018; Lotfi et al., 2020), remains a very important line of research to strengthen studies of brain criticality.

## Data Availability Statement

The raw data supporting the conclusions of this article will be made available by the authors, without undue reservation.

## Ethics Statement

Housing, surgical and recording procedures were in strict accordance with the CONCEA-MCTI, and was approved by the Federal University of Pernambuco (UFPE) Committee for Ethics in Animal Experimentation (23076.030111/2013-95, 12/2015 and 20/2020).

## Author Contributions

TC, MG-S, PC, and MC designed the study, interpreted the results, and wrote the manuscript. TC ran the simulations. TC and AF analyzed the data. TF, LA, TS, and NV obtained the experimental data. All authors read and approved the submitted version.

## Conflict of Interest

The authors declare that the research was conducted in the absence of any commercial or financial relationships that could be construed as a potential conflict of interest.

## Footnotes

^1^https://github.com/cortex-lab/phy
