# Supplementary material for: Subsampled Directed-Percolation Models Explain Scaling Relations Experimentally Observed in the Brain
Source: Front Neural Circuits. 2021 Jan 15;14:576727. doi: 10.3389/fncir.2020.576727 (PMC7843423; doi:10.3389/fncir.2020.576727)
Supplement: Supplementary file 1 [file Data_Sheet_1.PDF]

Supplementary Material

1 CV DISTRIBUTION AS A FUNCTION OF MODEL PARAMETERS

Here we illustrate the distribution of CV values as parameter values of the models are varied. In both cases, CV was estimated like in experimental data, i.e. for a subsampled number of units and during a finite window of time. Note that, as the net excitation decreases along the horizontal axes in Figure S1, the average values of CV initially increase, then saturate.

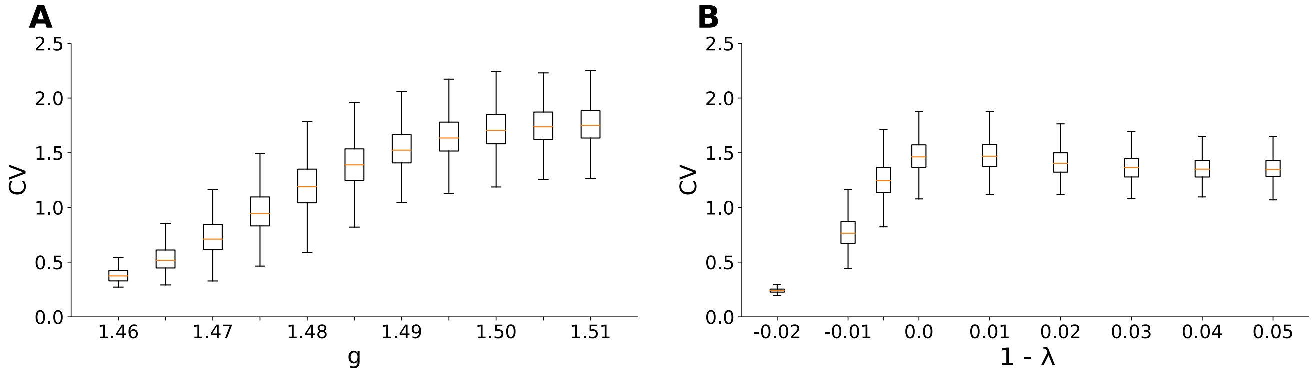

Figure S1. Boxplots of CV as a function of model parameters. **(A)** g for the spiking model with excitation and inhibition and **(B)** $1 - \lambda$ for the probabilistic cellular automaton model. In both cases, $w = 10$ s, just like for experimental data. **(A)** $n = 100$. **(B)** $n = 500$.

2 SCALING RELATION ANALYSIS IN EXPERIMENTAL DATA

In Figure 3A of the main text, we presented the group analysis of the experimental data for the right and left sides of Equation (4). As described in the Methods section, we analyzed the exponents τ, τ_t, and $1/(\sigma\nu z)$ for CV groups formed by $NB = 50$. This procedure was performed for the spike time series of each rat. In Figure S2, we show the results of the analysis of these exponents for each animal studied.

3 RESULTS FOR THE CELLULAR AUTOMATON MODEL

Simulations for the cellular automaton model described in Section 2.2 of the main paper yielded results similar to those obtained for the spiking model. Figure S3 shows the same plots as in Figures 2 and 3, with similar values of the exponents and of the spiking variability at which the scaling relation in Equation (4) is satisfied: at $CV_*^{CA-model} = 1.30 \pm 0.05$, $\tau_*^{CA-model} = 1.71 \pm 0.03$, $\tau_{t*}^{CA-model} = 1.94 \pm 0.03$ and $1/(\sigma\nu z)_*^{CA-model} = 1.33 \pm 0.02$.

Note also the same tendency of the model to reproduce the data for slightly supercritical values with the coupling parameter ranging from $1.00 \leq \lambda \leq 1.01$. Fluctuations around the critical point $\lambda_c = 1$ in parameter space are therefore in the range of 1%.

4 ROBUSTNESS WITH RESPECT TO TIME BIN

In Figure 4 of the main text, we explored how the exponents τ and τ_t depend on the number of sampled units n and the choice of two different bins for the analysis of avalanches: $\Delta t = \langle ISI \rangle$ and $\Delta t = 1$ ms. Here, we probed further the robustness of the results for experimental and subsampled model data in

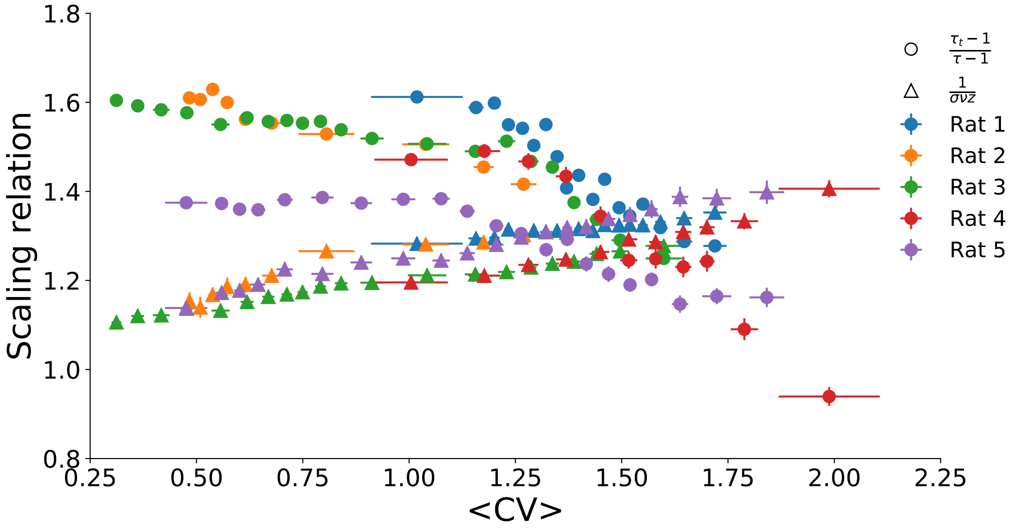

Figure S2. Right- and left-sides of Equation (4) as a function of the average CV for each rat evaluated. Through group analysis, we obtain that the scale relation is satisfied at the crossing in 1.3 ± 0.02 and at $CV_* \simeq 1.46 \pm 0.08$. We also highlight that all exponents obtained here satisfy Akaike's Information Criterion.

the evaluation of $\langle CV \rangle_*$, τ_*, and τ_{t*} that satisfy the criticality criterion (Equation 4) by assesssing their dependence on Δt (in multiples of $\langle ISI \rangle$) and the time window w used to evaluate CV.

As shown in Figures S4A and S4B, both for the experimental data and for the subsampled models, $\langle CV \rangle_*$, τ_*, and τ_{t*} decrease with the increase of the time bin Δt, a result which is consistent with those originally obtained by Beggs and Plenz (2003). In Figures S4C and S4D, $\langle CV \rangle_*$, τ_*, and τ_{t*} do not suffer great deviations, being largely insensitive to w.

5 FULL SAMPLING AND CV PARSING

Throughout the main text, we showed how the experimental results are reproduced when we impose subsampling around the critical point in models belonging to the MF-DP universality class (see Figures 3 and 4). Since we allowed g to vary around its critical value g_c, it is reasonable to ask whether these changes in the coupling parameter plus CV parsing would be sufficient ingredients to yield distorted exponents in the fully sampled model as well. To test these ideas, we performed CV parsing in the range of $1.47 \leq g \leq 1.55$ (supercritical to subcritical phase) considering all sites in the network (full sampling) and the results are shown in Figure S5. The scaling relation and the exponents obtained were completely different from those obtained experimentally, remaining close to MF-DP values. These results reinforce the idea that subsampling is crucial for the distortion of the exponents from MF-DP to those observed experimentally.

6 CV AS A PROXY OF CORTICAL STATE

It is known in the neuroscience literature that different levels of spiking variability are related to different cognitive states (Shadlen and Newsome, 1998; Harris and Thiele, 2011; de Vasconcelos et al., 2017). In urethane anesthetized brains, there is a slow modulation of the level of synchronization of the ongoing activity. From the experimental perspective, since we cannot ensure stationarity, it is preferable to use the minimum necessary time to calculate CV and define a cortical state. In the literature, a window of duration

$w = 10$ s is typically employed to estimate a cortical state (Gervasoni et al., 2004). Here we can evaluate, from the model perspective, if this value of w can already provide a good estimation of CV. In Figure S6, we evaluate the standard deviation σ_{CV} of CV as a function of the time used to estimate it. Note that a time bin of 20 s provides a better discrimination between the cortical states, saturating the decay of the standard deviation of CV for the experimental data. Despite the fact that a change in w does not impact the results (Figures S4C and S4D), in experiments one needs to compromise between a better statistical definition of CV and not mixing different states due to nonstationarity.

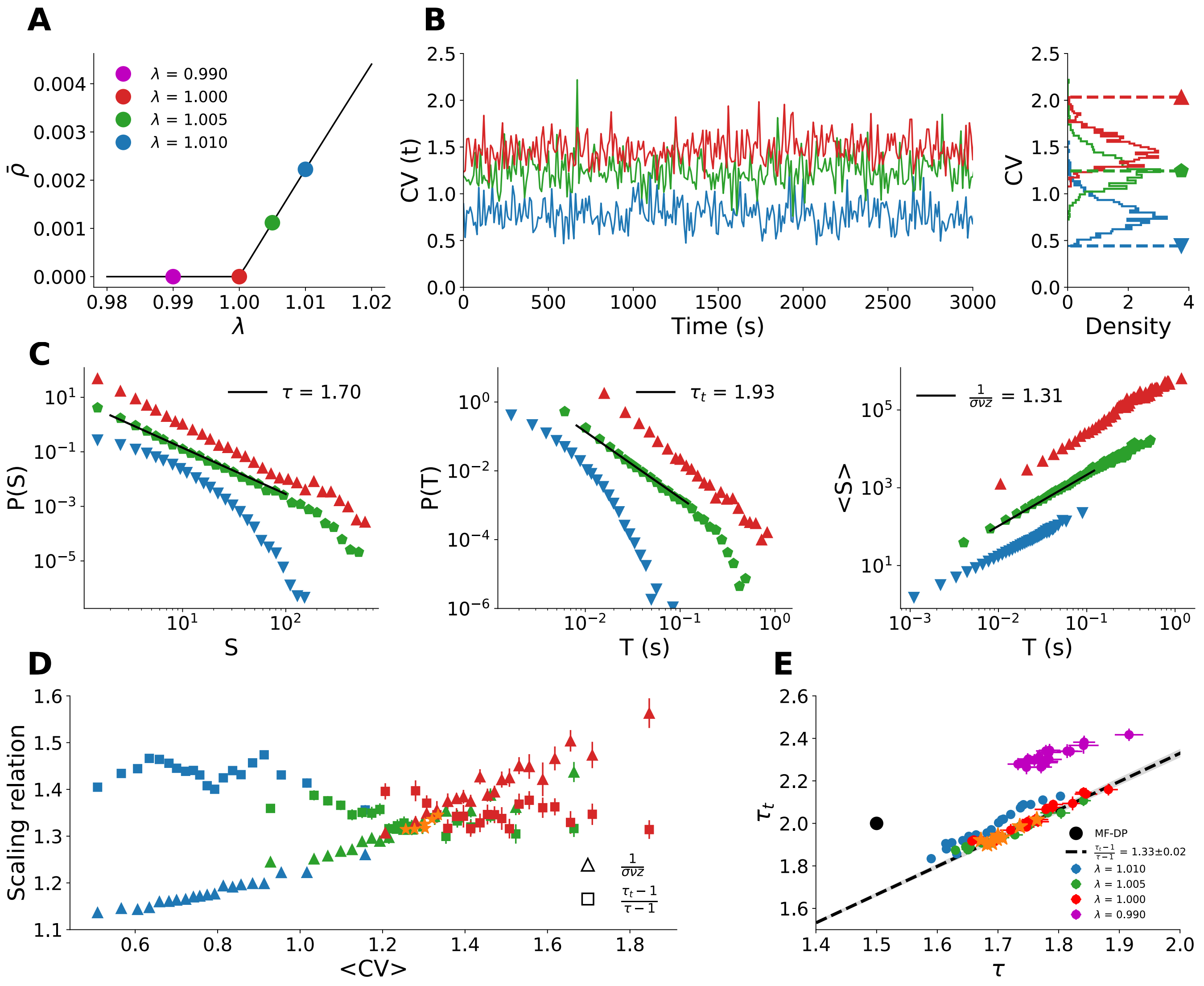

Figure S3. A probabilistic cellular automaton model with excitation only. **(A)** Stationary density of active sites as a function of the control parameter (branching ratio) λ for the fully sampled model with $N = 10^5$ (see Section 2.2 in the main paper). Points are simulations and lines are the linear expansion of the mean-field solution. All the remaining plots are for the subsampled model with $n = 500$. **(B)** CV time series and histogram around the critical point $\lambda_c = 1$. **(C)** Exponents τ, τ_t and $1/(\sigma\nu z)$ depend on λ. **(D)** Right- and left-hand sides of the scaling relation Equation (4) coincide around $CV_*^{CA-model} = 1.30 \pm 0.05$. (e) Spread of exponents τ and τ_t around the slope $1/(\sigma\nu z)_*^{CA-model} = 1.33 \pm 0.02$.

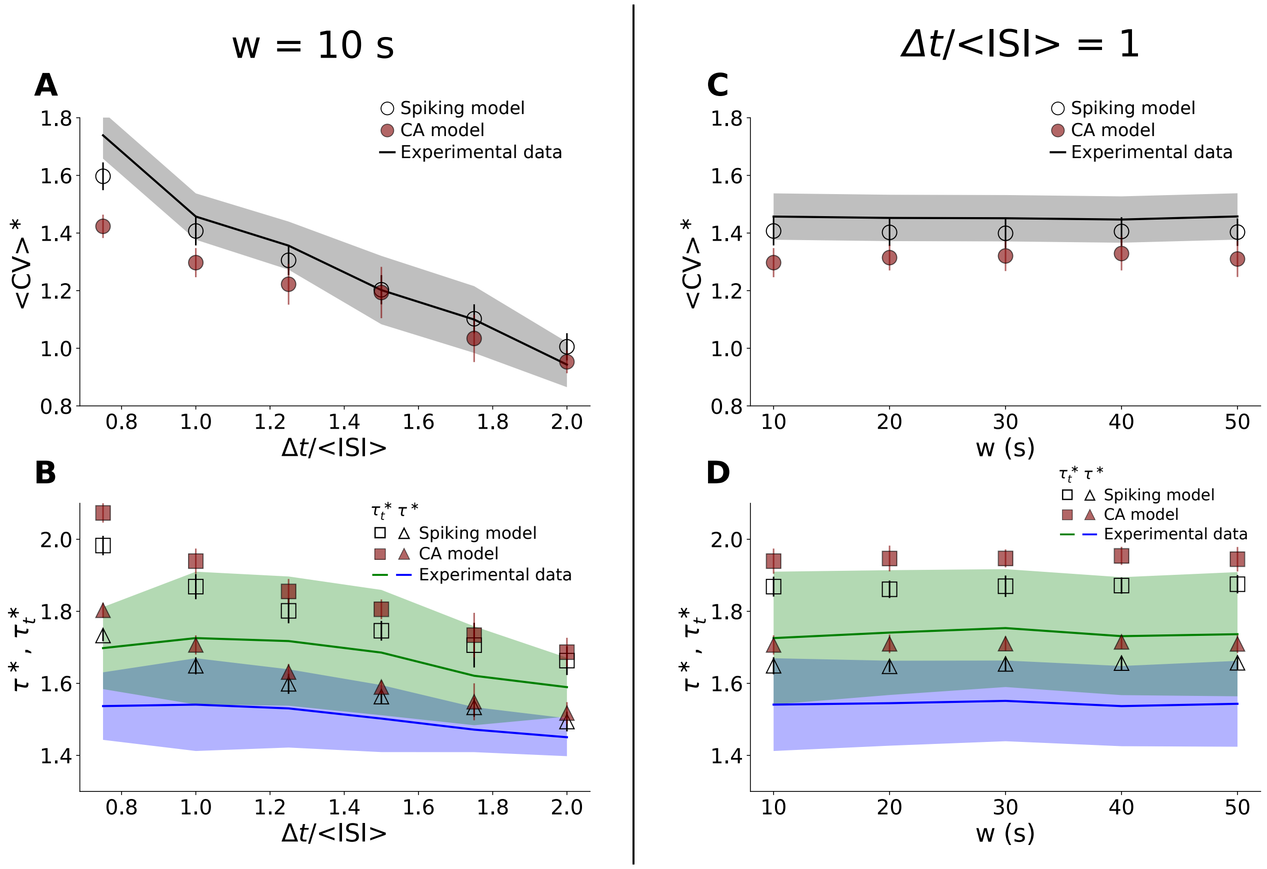

Figure S4. Dependence of $\langle CV \rangle_*$, τ_* and τ_{t*} on the temporal windows w and Δt. We compared the group analysis for the experimental data with results for the subsampled models ($n = 100$ for the spiking model and $n = 500$ for the cellular automaton model). For $w = 10$ s, we evaluated **(A)** $\langle CV \rangle_*$ and **(B)** τ_* and τ_{t*} at the point where Equation (4) is satisfied, varying the time bin Δt used to calculate avalanches. Next we fixed $\Delta t = \langle ISI \rangle$ and varied w, the time window to calculate CV, and we evaluated **(C)** $\langle CV \rangle_*$ and **(D)** τ_* and τ_{t*} at the point where Equation (4) is satisfied. We noticed that in all these scenarios, the results from the subsampled models follow the behavior of the experimental data. For the spiking model, g was varied from 1.47 to 1.50 and for the cellular automaton model, λ was varied from 1.00 to 1.01 (for both models, $N = 10^5$). In **(C)** and **(D)**, we use $NB = \{50, 25, 16, 12, 10\}$ for $w = \{10, 20, 30, 40, 50\}$ s to keep the total sampling time approximately the same (see Methods section in the main paper).

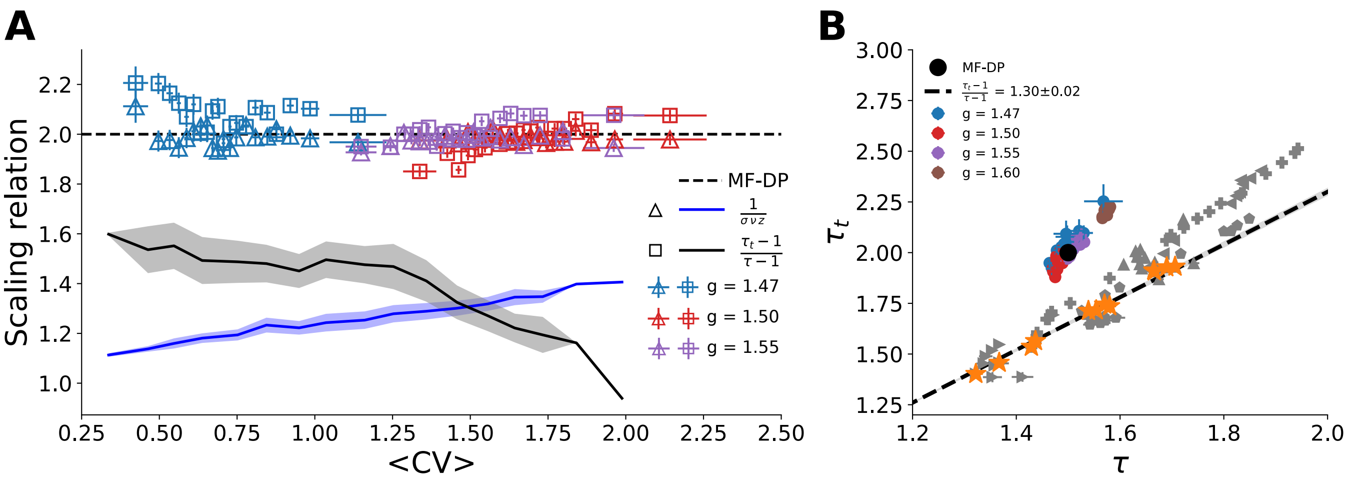

Figure S5. Results for full sampling ($N = 10^5$), CV parsing and $\Delta t = 1$ ms (points). **(A)** Scaling relation versus $\langle CV \rangle$. The shaded curves reproduce the group results of the experimental data (Figure 3A). **(B)** Scatter plot in the (τ, τ_t) plane. The gray points represent the exponents obtained from experimental data (as in Figure 3B).

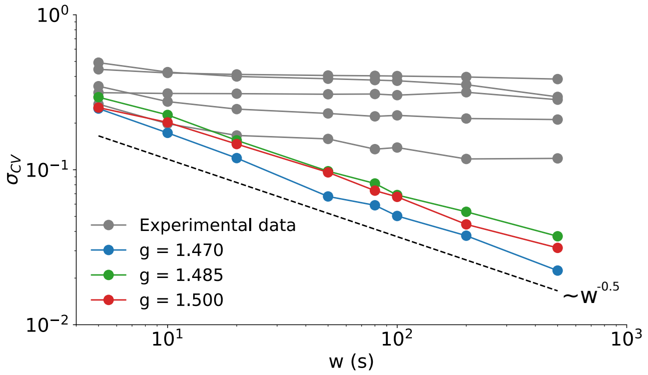

Figure S6. Dependency of the standard deviation (σ_{CV}) of the CV time series with the time window w used to estimate it. Each gray curve represents the result for each rat studied. The colored curves represent values of g of the subsampled spiking model with $n = 100$.

REFERENCES

Beggs, J. M. and Plenz, D. (2003). Neuronal avalanches in neocortical circuits. *J. Neurosci.* 23, 11167–11177

de Vasconcelos, N. A. P., Soares-Cunha, C., Rodrigues, A. J., Ribeiro, S., and Sousa, N. (2017). Coupled variability in primary sensory areas and the hippocampus during spontaneous activity. *Sci. Rep.* 7, 46077

Gervasoni, D., Lin, S., Ribeiro, S., Soares, E. S., Pantoja, J., and Nicolelis, M. A. L. (2004). Global forebrain dynamics predict rat behavioral states and their transitions. *J. Neurosci.* 24, 11137–11147

Harris, K. D. and Thiele, A. (2011). Cortical state and attention. *Nat. Rev. Neurosci.* 12, 509

Shadlen, M. N. and Newsome, W. T. (1998). The variable discharge of cortical neurons: implications for connectivity, computation, and information coding. *J. Neurosci.* 18, 3870–3896
